# Co‐created in vivo pharmacology practical classes using the novel organism *Lumbriculus variegatus*

**DOI:** 10.1002/prp2.1158

**Published:** 2023-12-08

**Authors:** Julanta J. Carriere, Nia A. Davies, Margaret R. Cunningham, Melisa J. Wallace, Aidan Seeley

**Affiliations:** ^1^ Swansea Worm Integrative Research Laboratory (SWIRL) Swansea University Medical School, Swansea University Swansea UK; ^2^ Strathclyde Institute of Pharmacy and Biomedical Sciences University of Strathclyde Glasgow UK

**Keywords:** animal models, co‐creation, histamine, invertebrates, student‐led, teaching

## Abstract

Co‐creation within higher education emphasizes learner empowerment to promote collaboration between the students and staff, enabling students to become active participants in their learning process and the construction of resources with academic staff. Concurrently, a diminishing number of higher education institutions offer in vivo practical classes, resulting in an in vivo skills shortage. To address this, and to actively engage students in their own learning, we describe the co‐creation of a student‐led drug trial using *Lumbriculus variegatus*. Under blinded conditions, final‐year undergraduate biomedical science students, under the tutelage of academic staff and fellow students, were involved in the co‐creation of an in vivo practical class to determine the effects of histamine and histamine receptor inverse agonists mepyramine and loratadine. Throughout this process, undergraduate‐ and masters‐level students played key roles in every aspect of practical delivery and data analysis. Herein, students demonstrated the test compounds, both in isolation and in combination, resulted in reduced stereotypical movements of *L. variegatus* (*p* < .05, *n* ≥ 6). 15% of students in the class responded to a feedback survey (*n* = 8) after the class. Students reported the class provided “*real life*” insights into in vivo research and enabled the development of hands‐on skills which would be useful in applying in their future careers. All students reported that they enjoyed the class with 25% (*n* = 2) reporting concerns about animal use in research, enabling useful discussions about animals in research. Moreover, these student‐led in vivo trials add to the pharmacological knowledge of *L. variegatus* promoting education‐led research.

## Graphical abstract

Diagrammatic representation of the workflow of the student‐led drug trials using *Lumbriculus variegatus*. (A) Drug compounds are selected by final year undergraduate students and masters‐level students. (B) Twenty‐four hours before the practical class, *L. variegatus* are isolated and placed on a 6‐well plate by these students. (C) Drug solutions are prepared on the day of the practical class and provided to the students under blinded conditions to test the effects on *L. variegatus* stereotypical movement with data then being recorded electronically. (D) Practical class attendees then adjust the experimental protocol based on their observations and upon competion of the class (E) enter their data onto cloud‐based spreadsheet with other experimental replicates for (F) blinded analysis and (G) graphing of data by final year undergraduate students and Masters‐level students. (H) Following graphing and analysis, the combined data can then be returned to students unblinded. Diagram created using images provided from Servier Medical ART.

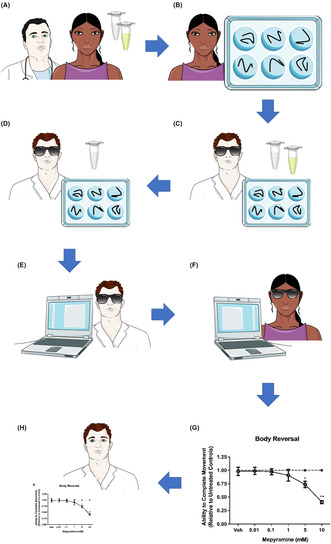

**Abbreviations**
DMSO: dimethyl sulfoxide
SBL: simulation‐based learning

## 1 INTRODUCTION

In just under two decades, the number of animals used for education and training in the United Kingdom has declined 89.7% from 5771 in 2001^1^ to 591 in 2020.^2^ While learned societies have highlighted the importance of continued in vivo skills training in education,^3^, ^4^ institutions are increasingly moving away from in vivo use, citing increased costs, a diminishing pool of trained staff, increased regulation, and ethical concerns.^5^, ^6^, ^7^ This approach has resulted in an in vivo skills gap as identified by The Association for the British Pharmaceutical Industry,^8^, ^9^ and, therefore, alternative routes for in vivo training of students are essential to ensure graduates have the required skills for the development of new medicines and therapeutics. The vast majority of scientists and the public agree that the reduction, refinement, and replacement (the 3Rs) of animals used in research are welcome and necessary.^10^ However, animal testing at some stage is still an essential part of the drug discovery process that cannot yet be replaced without sacrificing safety.^11^ To address this skills gap, many institutions have moved to simulation‐based learning (SBL) tools. SBL is an invaluable pedagogical tool that offers the opportunity to replace and demonstrate real experiences with guided experiences to replicate substantial aspects of real‐world techniques which can be linked to learning objectives.^12^ While many aspects of student learning outcomes can be met using virtual or remote practical classes ensuring student knowledge and understanding, such classes cannot fully replace the skills acquired through hands‐on practical training.^13^

While virtual laboratories can be useful in meeting learning outcomes, they do not fully recapitulate the complexity of conducting the work hands‐on.^14^ With the shift to virtual laboratories due to the pandemic, studies have highlighted the benefits of taking a hybrid approach to in vivo laboratory practicals, where implementation of pre‐laboratory simulation resources has been found to enhance student confidence for subsequent in‐person experimental work. A systematic review by Zhang et al.^14^ highlighted that the utilization of virtual laboratories is as effective as in‐person laboratories for learning concepts, but these do not enable the acquisition of practical skill sets. Additionally, students who undertake both virtual and in‐person animal work demonstrated preferences for hands‐on experiences.^15^ Furthermore, students who experience non‐simulation‐based practical classes report higher confidence and an increased understanding of the importance of in vivo research.^16^

Recently, we demonstrated that *Lumbriculus variegatus*, an aquatic Oligochaeta worm inhabiting shallow freshwater ponds, lakes and marshes,^17^ has the potential for use within in vivo pharmacology education.^18^ As an invertebrate, *L. variegatus* is exempt from the Animal (Scientific Procedures) Act 1986 and, therefore, offers the opportunity for utilization within education settings. Unlike conventional in vivo organisms, *L. variegatus* is low‐cost and exempt from much of the regulation and ethical challenges that are prohibitory to traditional in vivo practical classes.^5^, ^6^

*Lumbriculus variegatus* displays two characterized stereotypical movements whereby tactile stimulation of the anterior region results in retraction and the reversal of the body position while touching the tail elicits helical swimming movements.^17^ These movements are easily quantifiable without the requirement of any specialist equipment and can be altered by exposure to drug compounds with diverse pharmacodynamic properties.^17^, ^18^, ^19^, ^20^ As such, quantification of *L. variegatus* stereotypical movements in the presence and absence of drug compounds enables the inclusion of practical in vivo behavioral pharmacology within a teaching environment.

Many traditional practical classes are aimed at students achieving a specific result with expected outcomes known in advance and designed without student collaboration and have no avenue for co‐creation. Co‐creation learning refers to students being actively involved with the design and development of educational practices.^21^ Co‐creation of learning environments enables students to collaborate with educators in the design of their own learning experience with the educator becoming a co‐student, accepting students as knowledgeable participants and partners in their learning experience.^21^, ^22^ Studies have demonstrated that co‐creation facilitates learning and can improve students' skills and knowledge acquisition.^23^, ^24^ Co‐creation can present as challenging for both students and teachers; however, these challenges can be addressed by involving students early to build their confidence and developing strategies to support student engagement.^25^ Herein, we present the first documented implementation of co‐creation within a biomedical sciences programme within in vivo practical classes.

We aimed to deliver a novel whole animal practical class to undergraduate students under blinded conditions to demonstrate to students how blinding, the process of withholding information about the assigned treatment from individuals,^26^ is used in experiments and to allow students to engage directly with in vivo research and experimental design. The effects of all compounds used within this practical class were not previously tested in *L. variegatus* to allow students to directly add to the pharmacological knowledge of this novel organism. The practical was developed, prepared and delivered with undergraduate and masters‐level students with a co‐creation learning objective which determines what effect, if any, the blinded compounds had on *L. variegatus* behavior. Using our novel stereotypical movement assay,^18^ students examined the effects of three distinct compounds which target histamine receptors; the endogenous histamine receptor ligand, histamine,^27^ and the histamine H_1_ receptor inverse agonists, mepyramine ^28^ and loratadine.^29^

We demonstrate that students are capable of utilizing *L. variegatus* in a practical class due to this organism being a technically straightforward yet effective tool for the teaching of in vivo behavioral pharmacology. Students generated robust and reproducible data thus adding to the knowledge of histamine receptor pharmacology within *L. variegatus*. Moreover, students self‐reported that they enjoyed contributing to the study and that they felt it would be useful in their future careers. Herein, we present the findings and student feedback from the first student‐led in vivo drug trial under blinded conditions.

## 2 METHODS

### 2.1 *Lumbriculus variegatus* culture

*Lumbriculus variegatus* were cultured in artificial pond water as previously described.^18^ Briefly, *L. variegatus* were cultured in artificial pond water for a minimum of 3 months before experimentation to limit variation within the cultures. Continuous aeration and water filtration were achieved by commercial air stones and aquarium filters, respectively. The artificial pond water was changed weekly and cultures were fed TetraMin flakes and 10 mg/L spirulina weekly, subject to a 16:8‐h light–dark cycle and stored at room temperature (18–21°C). Once *L. variegatus* were added to the aquarium, no attempts were made to monitor or adjust the pH of the artificial pond water.

### 2.2 Solutions and reagents

Final year undergraduate project students and masters‐level students were involved in the selection process of drug compounds to be tested. Histamine, mepyramine and loratadine were obtained from Sigma‐Aldrich (Dorset, United Kingdom) and dissolved in artificial pond water. A 12 mM loratadine stock solution was made by dissolving in 100% dimethyl sulfoxide (DMSO) (Sigma‐Aldrich, Dorset, United Kingdom) and subsequent dilution in artificial pond water to give a final DMSO concentration of 0.5% and a maximum final loratadine concentration of 60 μM. All drug compounds were made up on the day of use and blinded to students throughout experiments and analysis.

### 2.3 Student‐led drug trial practical

This student‐led in vivo drug trial was delivered as a practical class to first year undergraduate biomedical science students based at Swansea University Medical School, United Kingdom. The practical class was delivered in collaboration with educators, technicians, final‐year undergraduate project students and masters‐level students.

Twenty‐four hours before the practical class, one *L. variegatus* worm was placed in each well of a Cellstar® 6‐well plate (Greiner Bio‐One) containing 4 mL of artificial pond water. Individual worms used in experiments were randomly selected, lacked any obvious morphological defects, and ranged from 2 to 8 cm in length as per previous studies.^18^, ^19^ Plates were kept at room temperature and subjected to a 16:8‐h light–dark cycle until used.

On the day of the practical class, drug solutions were prepared as previously described, and students were arranged into pairs. Each group received three 6‐well plates with *L. variegatus* and were provided with two different blinded drug solutions. All groups were provided with 10 mM histamine and either 10 mM mepyramine or 60 μM loratadine in artificial pond water under blind conditions.

Students conducted the *L. variegatus* stereotypical movement assay for the blinded drug compounds using the methodology previously described^18^ under the tutelage of final year undergraduate project students and masters‐level students, with educators and technicians present throughout the class for additional support.

Briefly, students conducting the assay replaced the artificial pond water and the baseline ability of the worm to perform stereotypical behaviors was tested and recorded to give the baseline measurements. This was achieved by alternately stimulating the anterior or posterior regions of *L. variegatus* with a 20 to 200 μL plastic pipette tip, five times per end, with a 5–10 s interval between stimuli. Students then objectively scored the stereotypical movements as 1 = No movement, 2 = Incomplete Stereotypical movement, and 3 = Full Stereotypical Movement.

The artificial pond water was then removed and immediately replaced with drug solution or vehicle controls (artificial pond water only or 0.5% DMSO in artificial pond water). *Lumbriculus variegatus* were incubated with the drug solution or vehicle control for 10 min and students then re‐tested the ability of *L. variegatus* to respond to tactile stimulation (drug exposure). Drug solutions and vehicle controls were then aspirated from the wells and, to remove any latent drug or vehicle residue, fresh pond water was added and then immediately aspirated and then replaced with 4 mL fresh artificial pond water. These worms were then re‐tested at 10 min (Rescue 10 min) by class attendees during the practical class and final year undergraduate project students and masters‐level students tested the behaviors 24 h (Rescue 24 h) post drug or vehicle control treatment.

Additionally, students measured the impact of administering drugs in combination on the stereotypical movement of *L. variegatus*. To enable co‐creation of the in‐class protocol, students were polled in class by raising their hands on which drug they wished to administer first and at what fixed concentration. In this instance, students selected to administer 1 mM histamine for 10 min and then expose *L. variegatus* to 0–10 mM mepyramine or 0–60 μM loratadine for 10 min before conducting stereotypical movement assays.

Decomposition, as determined by visible tissue degeneration and whole‐organism tissue pallor, at assay endpoints was the main indicator of lethal toxicity. *Lumbriculus variegatus* were euthanised at assay endpoints by rapid submersion in 70% ethanol.

**FIGURE 1:**
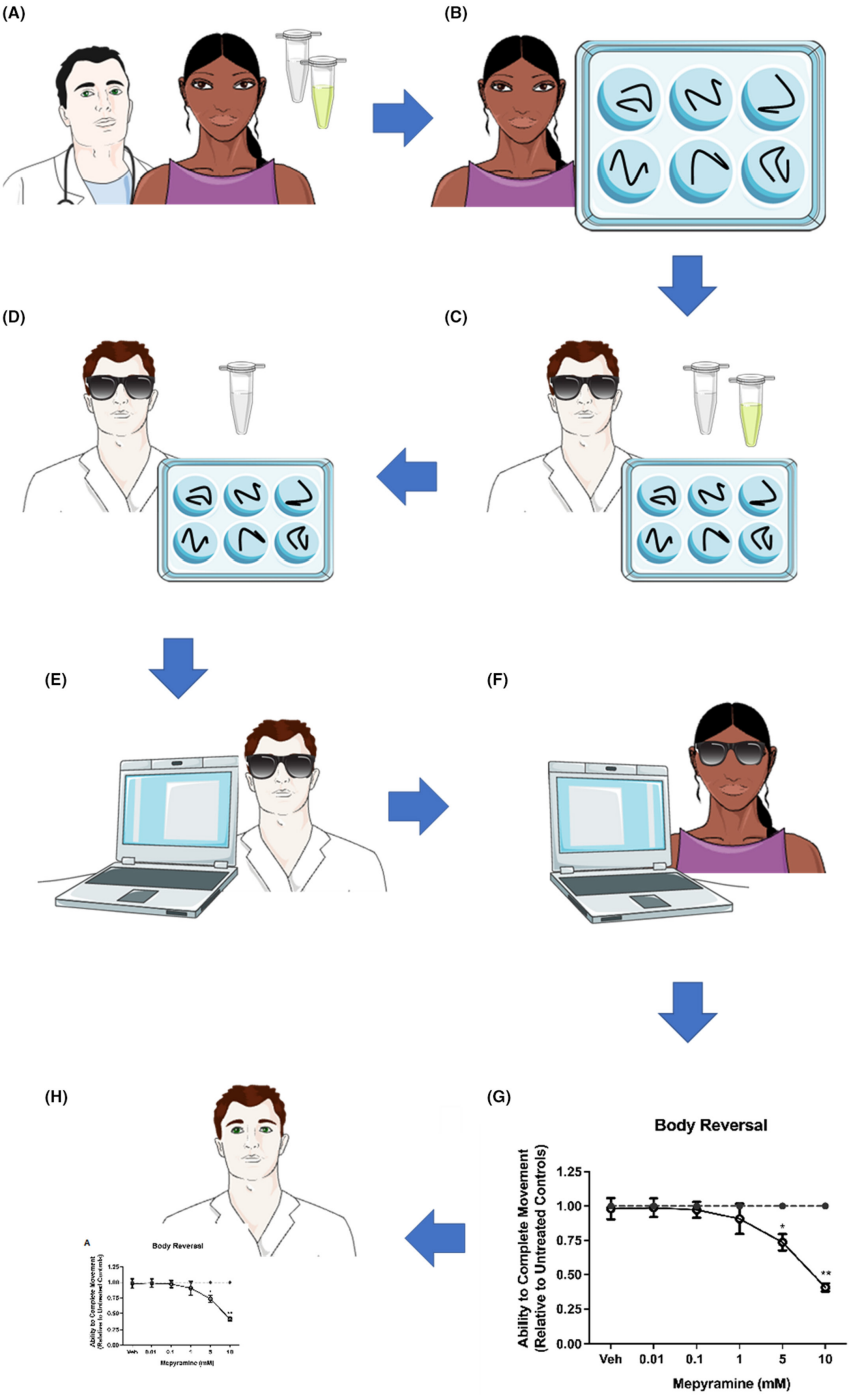
Diagrammatic representation of the workflow of the student‐led drug trials using *L. variegatus*. (A) Drug compounds are selected by final year undergraduate students and masters‐level students. (B) Twenty‐four hours before the practical class, *L. variegatus* are isolated and placed on a 6‐well plate by these students. (C) Drug solutions are prepared on the day of the practical class and provided to the students under blinded conditions to test the effects on *L. variegatus* stereotypical movement with data then being recorded electronically. (D) Practical class attendees then adjust the experimental protocol based on their observations and upon competion of the class (E) enter their data onto cloud‐based spreadsheet with other experimental replicates for (F) blinded analysis and (G) graphing of data by final year undergraduate students and masters‐level students. (H) Following graphing and analysis, the combined data can then be returned to students unblinded. Diagram created using images provided from Servier Medical ART.

Students reported their data via a cloud‐based spreadsheet and data were subsequently collated, and graphed using GraphPad Prism 9 by final year undergraduate project students and masters‐level students. Unblinded results were disseminated to the students after the practical class, presented herein, and analyzed data subsequently formed part of final year project students' dissertations. A summary of the practical class workflow is shown in Figure 1.

### 2.4 Statistical analysis

The sample size for each assay and treatment was ≥6 worms. Data are displayed as the mean ± standard error of the mean for each data set and are relative to the untreated control conditions (baseline). Values for each behavioral measurement were compared to the baseline for each *L. variegatus* per condition and data are expressed as a ratio of the movement score whilst in treatment relative to baseline. Data were analyzed by final year undergraduate project students and masters‐level students under blinded conditions. Drug exposure conditions were compared to baseline by paired non‐parametric two‐tailed *t*‐test, and 10‐min and 24‐h rescue time points were compared to baseline by two‐way ANOVA with Dunnett's post‐test. Statistical analysis was performed in GraphPad Prism 9, and *p* < .05 was the threshold for statistical significance.

### 2.5 Student feedback

Following completion of the practical class, all students who attended the class were asked to provide anonymous qualitative feedback on the practical class. This was done using an online survey through Microsoft Forms which was emailed to the students directly. Students were asked if they had any feedback, comments, or suggestions for the *L. variegatus* practical class. Final‐year undergraduate project students and masters‐level students were also asked for any comments or feedback on the class.

### 2.6 Nomenclature of targets and ligands

Key protein targets and ligands in this article are hyperlinked to corresponding entries in http://www.guidetopharmacology.org, the common portal for data from the IUPHAR/BPS Guide to PHARMACOLOGY,^30^ and are permanently archived in the Concise Guide to PHARMACOLOGY 2019/20.^31^

## 3 RESULTS

### 3.1 Behavioral response to histamine

**FIGURE 2:**
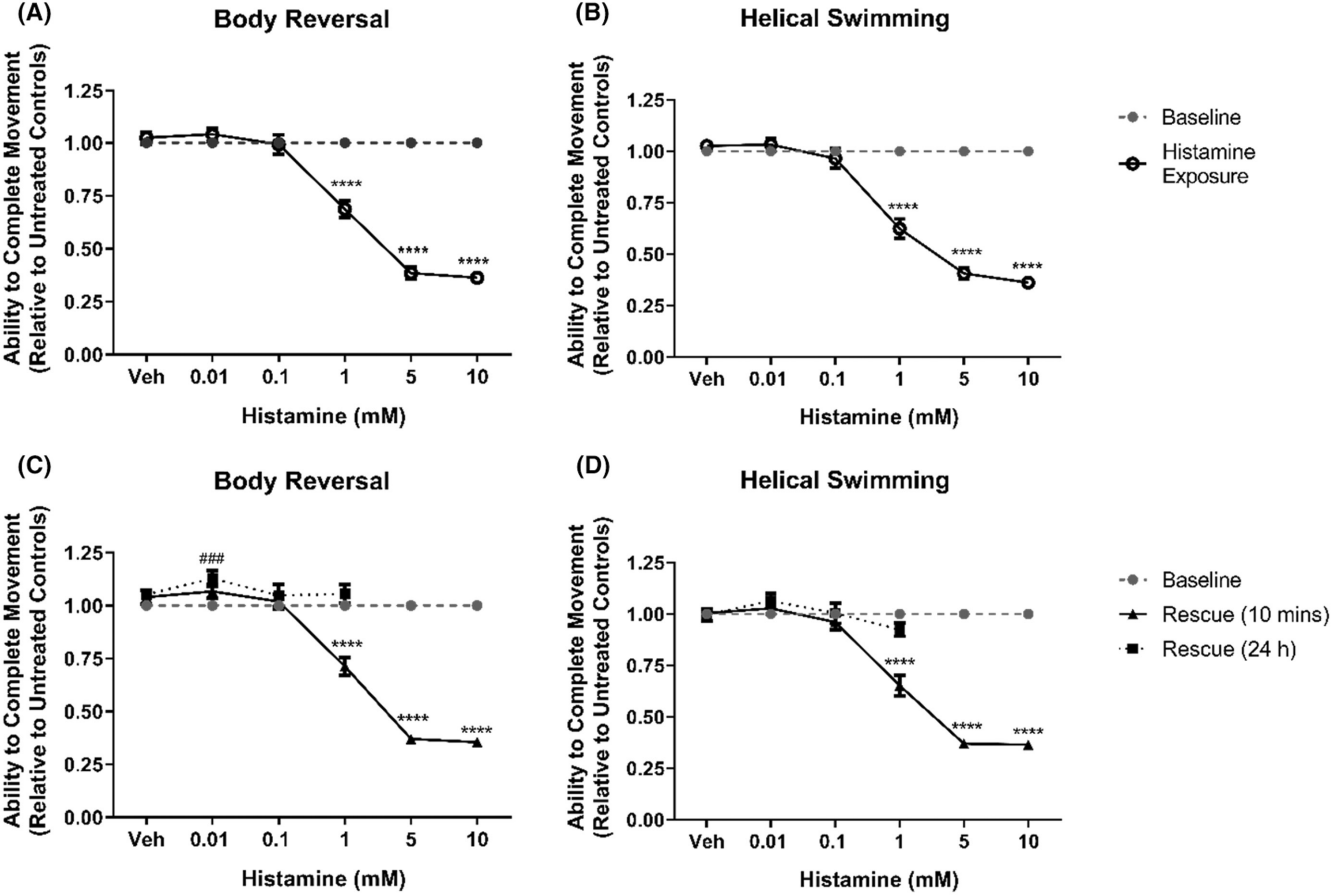
The effects of histamine on *Lumbriculus variegatus* behavior. *Lumbriculus variegatus* were exposed to histamine (0–10 mM) and tested for the ability of tactile stimulation to elicit (A) body reversal or (B) helical swimming. Histamine was then removed and after 10 min in artificial pondwater the ability of *L. variegatus* to perform (C) body reversal and (D) helical swimming after 10 min and 24 h. Data are expressed as a ratio of the movement score after exposure relative to the movement score at baseline. Error bars represent the standard error of the mean, *n* = 16 for each concentration. Veh: Artificial pondwater. ### *p* < .01, **** *p* < .0001, where * refers to statistical significance between baseline and histamine exposure or baseline and rescue (10 min), and # refers to statistical significance between baseline and rescue (24 h).

Histamine, the endogenous agonist for histamine receptors and a major neurotransmitter present in both vertebrates and invertebrates,^27^ was shown to inhibit *L. variegatus* stereotypical movements. Our students reported significant inhibition after 10 min of exposure to 1–10 mM histamine for both body reversal (*p* < .0001, Figure 2A) and helical swimming (*p* < .0001, Figure 2B), with effects persisting 10 min after removal for both movement swimming (*p* < .0001, Figure 2C,D). Twenty‐four hours after exposure, it was observed that 5 mM and 10 mM histamine resulted in lethal toxicity in all organisms tested (*n* = 16, Figure 2C,D).

### 3.2 Behavioral response to mepyramine

**FIGURE 3:**
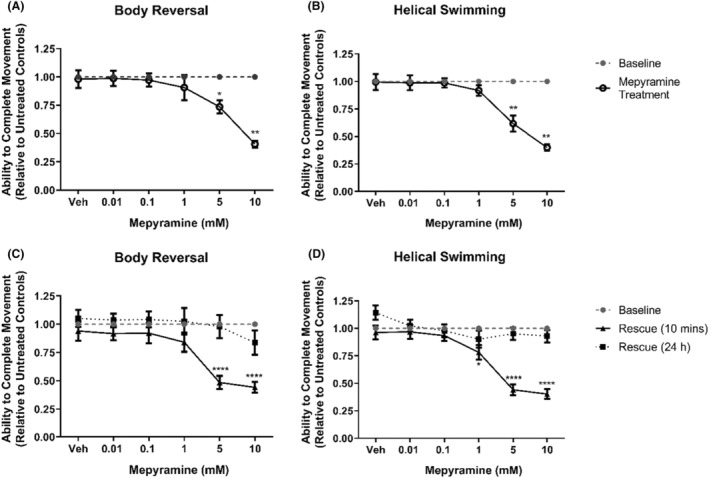
The effects of mepyramine on *Lumbriculus variegatus* behavior. *Lumbriculus variegatus* were exposed to mepyramine (0–10 mM) and tested for the ability of tactile stimulation to elicit (A) body reversal or (B) helical swimming. Mepyramine was then removed and after 10 min in artificial pondwater the ability of *L. variegatus* to perform (C) body reversal and (D) helical swimming after 10 min and 24 h. Data are expressed as a ratio of the movement score after exposure relative to the movement score at baseline. Error bars represent the standard error of the mean, *n* = 8 for each concentration. Veh: artificial pondwater. * *p* < .05, ** *p* < .01, **** *p* < .0001.

Our students found that the histamine H_1_ receptor inverse agonist mepyramine^28^ significantly inhibited both body reversal and helical swimming at 5 and 10 mM (*p* < .05, Figure 3A,B), with both movements remaining inhibited at 5 and 10 mM after the removal of mepyramine and incubation in artificial pond water (*p* < .0001, Figure 3C). Interestingly, the emergence of inhibition of helical swimming at 1 mM 10 min after removal was also observed (*p* = .0117, Figure 3D).

Mepyramine exhibited no lethal toxicity and 24 h post‐exposure to mepyramine, the ability to perform both stereotypical movements was shown to be indistinguishable from baseline conditions (*p* > .05, Figure 3C,D).

### 3.3 Behavioral response to loratadine

**FIGURE 4:**
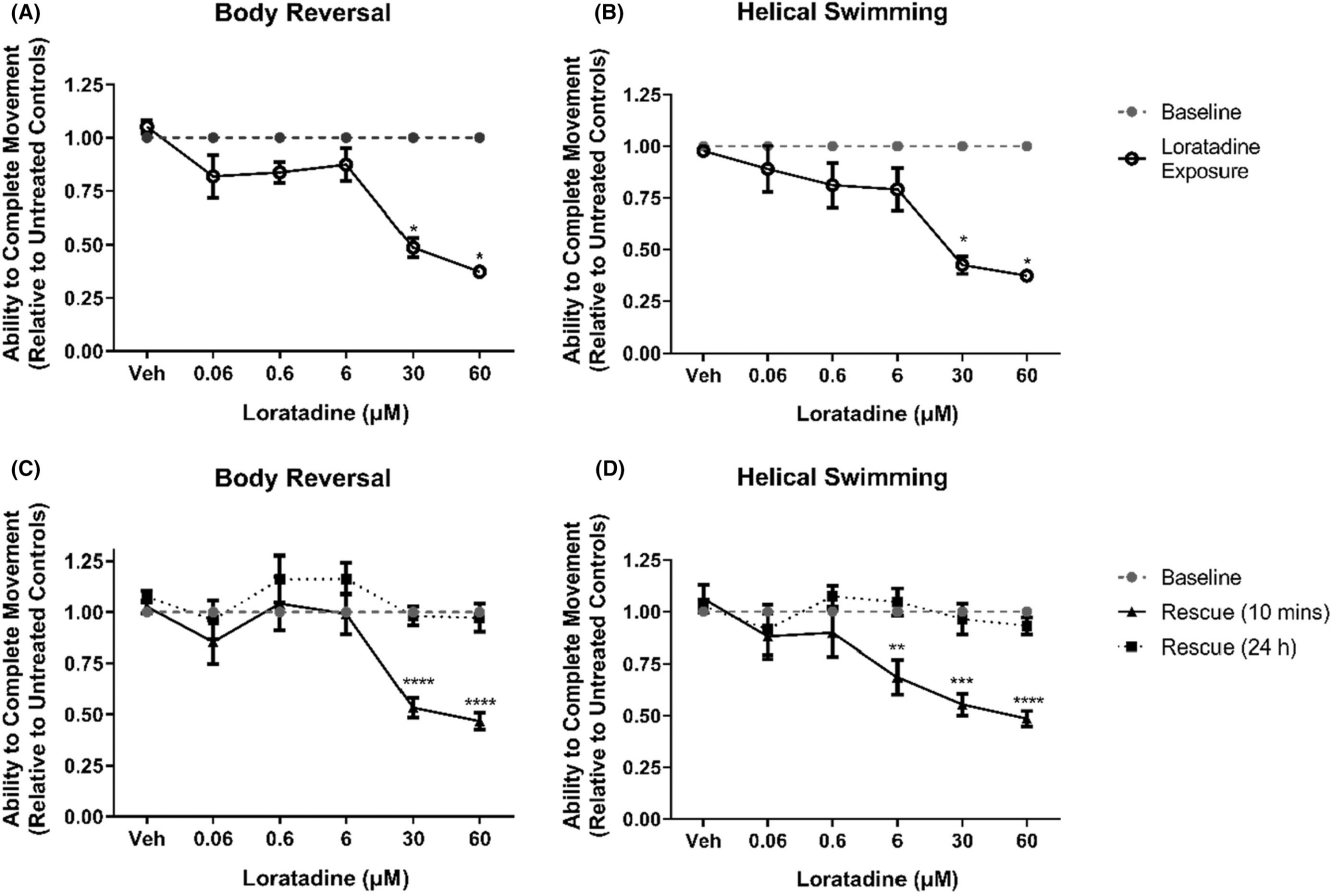
The effects of loratadine on *Lumbriculus variegatus* behavior. *Lumbriculus variegatus* were exposed to loratadine (0–60 μM) and tested for the ability of tactile stimulation to elicit (A) body reversal or (B) helical swimming. Loratadine was then removed and after 10 min in artificial pondwater the ability of *L. variegatus* to perform (C) body reversal and (D) helical swimming was tested after 10 min and 24 h. Data are expressed as a ratio of the movement score after exposure relative to the movement score at baseline. Error bars represent the standard error of the mean, *n* = 6 for each concentration. Veh: 0.5% DMSO in artificial pondwater. * *p* < .05, ** *p* < .01, *** *p* < .001, **** *p* < .0001.

Similar to mepyramine, loratadine is a histamine H_1_ receptor inverse agonist.^29^ Loratadine exhibited inhibitory functions for both stereotypical movements at 30 and 60 μM (*p* < .05, Figures 3A,B). After the removal of loratadine and incubation in artificial pond water for 10 min, it was observed that the ability of *L. variegatus* to perform stereotypical movements remained inhibited (*p* ≤ .0002, Figure 4C). Similar to mepyramine, a delayed effect was observed on helical swimming following loratadine removal. 10‐min after the removal of loratadine and incubation in artificial pond water, exposure to 6 μM loratadine exposure resulted in significant inhibition of helical swimming (*p* = .0098, Figure 4D). After 24 h in drug‐free artificial pond water, all *L. variegatus* were able to perform both body reversal and helical swimming movements at a level indistinguishable from baseline conditions (*p* > .05, Figure 4C,D).

### 3.4 Behavioral response to histamine and mepyramine or loratadine

Students co‐created the protocol for administering drugs in combination on the stereotypical movement of *L. variegatus*. Students self‐selected to administer 1 mM histamine for 10 min and then expose *L. variegatus* to 0–10 mM mepyramine or 0–60 μM loratadine for 10 min.

**FIGURE 5:**
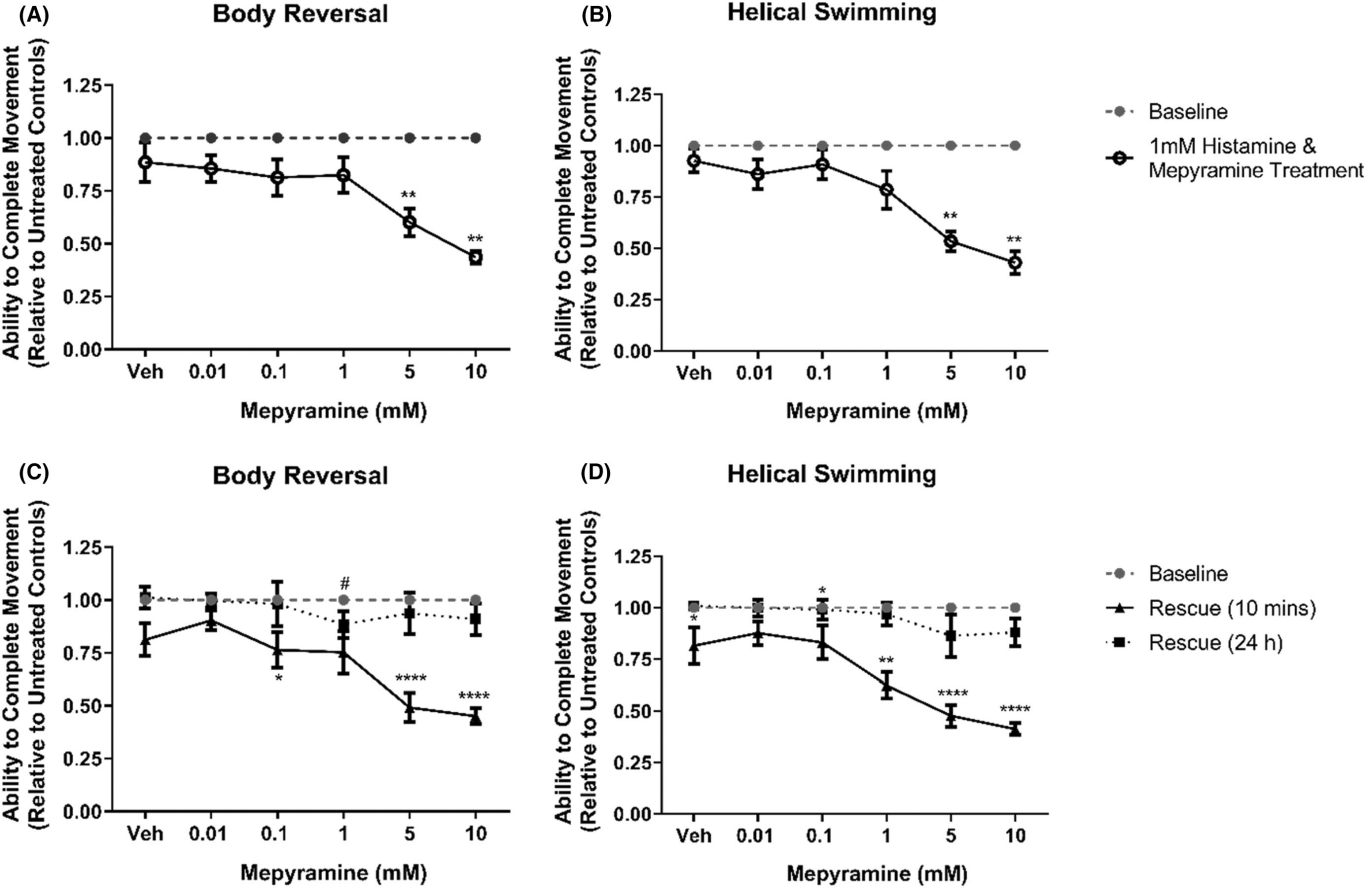
The effects of mepyramine treatment after histamine exposure on *Lumbriculus variegatus* behavior. All *L. variegatus* were exposed to 1 mM histamine for 10 min before being washed in artificial pondwater. *Lumbriculus variegatus* were treated with mepyramine (0–10 mM) for 10 min before being tested for the ability of tactile stimulation to elicit (A) body reversal or (B) helical swimming. Mepyramine was then removed and after 10 min in artificial pondwater the ability of *L. variegatus* to perform (C) body reversal and (D) helical swimming after 10 min and 24 h. Data are expressed as a ratio of the movement score after exposure relative to the movement score at baseline. Error bars represent the standard error of the mean, *n* = 9 for each concentration. Veh: artificial pondwater. */*# p* < .05, ** *p* < .001, **** *p* < .0001, where * refers to statistical significance between baseline and mepyramine exposure or baseline and rescue (10 min), # refers to statistical significance between baseline and rescue (24 h).

Administration of 1 mM histamine before mepyramine (0–10 mM) had inhibitory effects on both stereotypical movements at 5–10 mM (*p* < .01, Figure 5A,B). After the removal of mepyramine, it was observed that 0.1 mM mepyramine significantly inhibited body reversal (*p* = .0414, Figure 5C) and helical swimming (*p* = .0337, Figure 5D), as well as inhibiting these movements at 5–10 mM (*p* < .0001, Figure 5C,D).

**FIGURE 6:**
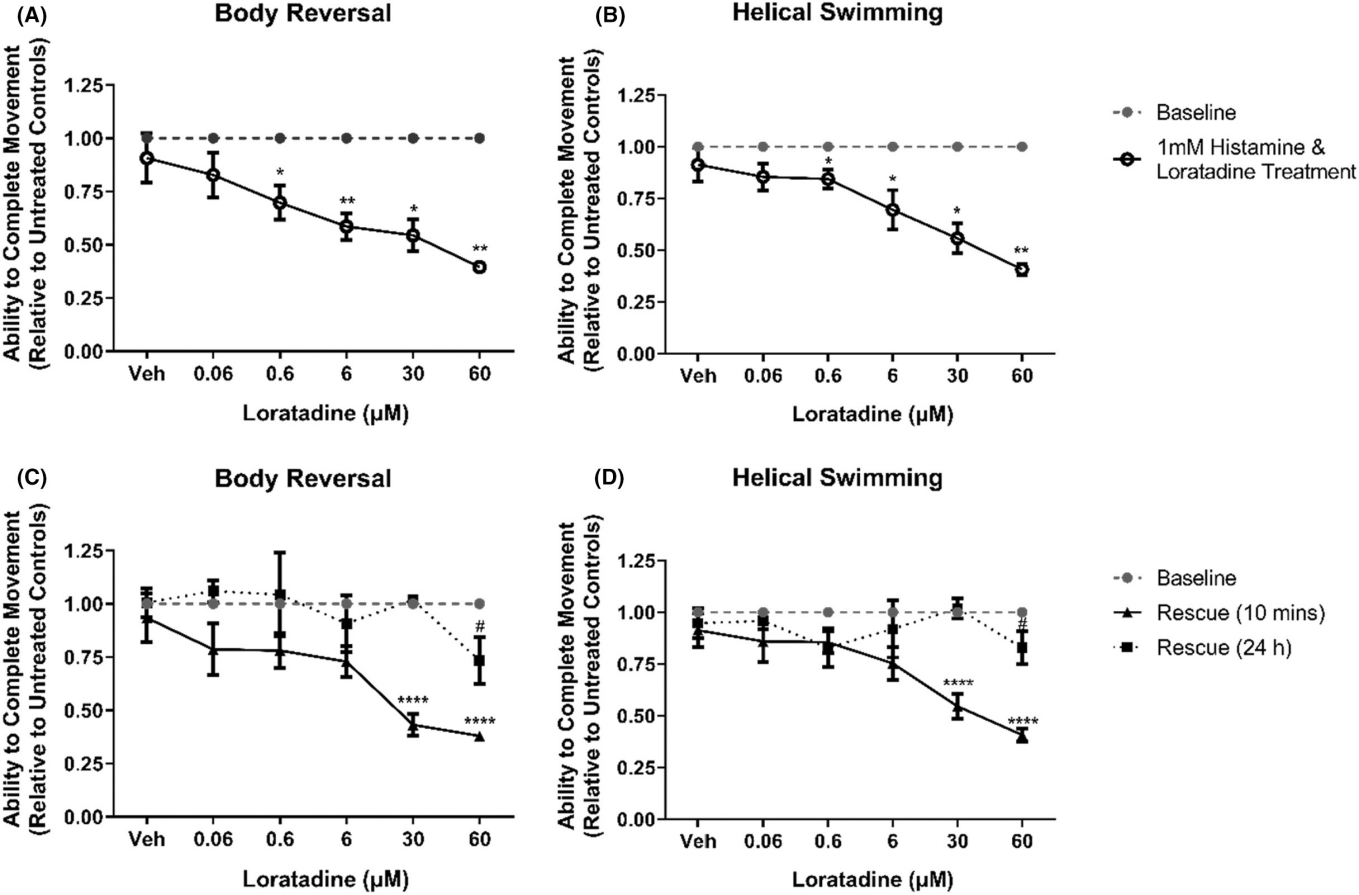
The effects of loratadine treatment after histamine exposure on *Lumbriculus variegatus* behavior. All *L. variegatus* were exposed to 1 mM histamine for 10 min before being washed in artificial pondwater. *Lumbriculus variegatus* were treated with loratadine (0–60 μM) for 10 min before being tested for the ability of tactile stimulation to elicit (A) body reversal or (B) helical swimming. Loratadine was then removed and after 10 min in artificial pondwater the ability of *L. variegatus* to perform (C) body reversal and (D) helical swimming after 10 min and 24 h. Data are expressed as a ratio of the movement score after exposure relative to the movement score at baseline. Error bars represent the standard error of the mean, *n* = 16 for each concentration. Veh: 0.5% DMSO in artificial pondwater. */*# p* < .05, ** *p* < .01, **** *p* < .0001, where * refers to statistical significance between baseline and histamine exposure or baseline and rescue (10 min), and # refers to statistical significance between baseline and rescue (24 h).

Administration of 1 mM histamine before loratadine (0–60 μM) inhibited stereotypical movements at doses ≥0.6 μM (*p* < .05, Figure 6A,B). Effects persisted at 30 μM and 60 μM after the removal of loratadine for 10 min for body reversal (*p* < .0001, Figure 6C) and helical swimming (*p* < .0001, Figure 6D). Long‐term effects were observed 24 h after exposure with body movements being inhibited after 1 mM histamine and 60 μM loratadine exposure (*p* < .05, Figure 6C,D).

### 3.5 Student feedback

All students (*n* = 54) who undertook the practical class were asked to provide qualitative feedback on the practical class using an anonymous online survey. There was a response rate of 15% (*n* = 8) with students stating:

I really enjoyed this practical, I think it was fun, engaging and gave me an insight into more real‐life research which is really interesting

It was a very enjoyable experience overall and was great to get some hands‐on experience with regards to in vivo research. This will come in very useful when applying for Masters/PhD

Although I personally am uncomfortable with the idea of animal research, I really enjoyed this experience and I am grateful to have had the rare opportunity to partake in an in vivo study

It was the most brilliant practical we ever had

I would propose using more computers to input the data

The input of the data was straightforward and simple and was nice to see the data calculations next to the data as well. It was also interesting to see which drugs we had administered after the practical data had been analysed

The key themes from the qualitative feedback obtained from undergraduate students undertaking the practical class were the insights into conducting “*real life*” research allowing for contribution to in vivo research while gaining hands‐on skills. Students reflected that these skills will be useful in applying for postgraduate studies such as at masters/PhD level.

Feedback from the final year undergraduate project students and masters‐level students who were involved in delivering the practical class found involvement with the class helped develop their understanding of experimental design and preparation while validating their knowledge and allowing the generation of data for their individual dissertation projects. Feedback from these students included:

These sessions supported my final year project as the data generated was used towards my results

I was involved in the decision‐making process when deciding the drugs/compounds to use. Discussions were led through knowledge & skills we gained when conducting our final year projects

Setting up the practical gave me a good understanding of the importance of experimental preparation and design

It was great experience to support other students in a teaching laboratory environment and helped to validate my knowledge

## 4 DISCUSSION

### 4.1 Co‐creation using *Lumbriculus variegatus*

Herein, we demonstrate that *L. variegatus* can be used to deliver in vivo practical classes to undergraduate students, with students directly contributing to the knowledge basis of the effects of pharmacological compounds on this organism. This blinded in vivo student‐led drug trial illustrates the applicability of *L. variegatus* as a novel organism for in vivo education and actively engages students in research while enabling students to engage in co‐creation which has been shown to increase learning.^25^

This class allowed students to develop key practical skills for biomedical laboratory work, such as drug dilutions, effective time management and teamwork skills as well as experimental design while addressing core concepts of dose–response relationships, drug reversibility and drug toxicity.

As part of the design of this practical class, students were provided with blinded compounds and instructed to perform the stereotypical movement assay on the *L. variegatus* as previously described.^18^ As students were blinded this reduced the risk of expectation influencing findings in research, leading to biased results. As these drugs were untested within *L. variegatus* previously, this is a more ethical use of these organisms as students were directly answering a research question to gain new knowledge rather than attempting to replicate a known experimental endpoint.

This co‐creation approach means that students were directly involved from the development of the experimental protocol to reporting on what they have observed. This form of co‐created teaching encourages an open exchange of ideas, and developing relationships between staff and students where education is done *with* students.^32^

Additionally, this practical class engages students with in vivo measurements and scoring, data recording and interpretation, and statistical analysis while implementing other key concepts such as experimental blinding and the 3Rs within a practical setting. Using our stereotypical movement assay^18^ under blinded conditions, students were capable of measuring the dose‐dependent effects of histamine, mepyramine and loratadine on the ability of *L. variegatus* to perform body reversal and helical swimming movements. Moreover, data generated from the practical class were integrated into final year projects to further utilize the data generated through this in vivo practical and making further use of this organism for education and research purposes.

Moreover, when asked to provide qualitative feedback, all respondents (*n* = 8) reported that they enjoyed the class; however, there was no specific feedback on the co‐created aspects in the feedback, but this was not explicitly requested in the feedback questionnaire. Importantly, this feedback highlights that students actively engaged with the class and, when provided with learning objectives through co‐creation, students were encouraged to actively contribute to the practical class set‐up, protocol and analysis, as well as to *L. variegatus* research more broadly in the first student‐led in vivo drug trial. Student feedback does raise some important logistical aspects of delivery of the practical, specifically with the data entry aspects of the class whereby sufficient access to computers is required to ensure timely and effective data entry, with increased computers available in future iterations of the class. Moreover, students engaged in the refinement of the experimental design, with students reporting after the practical class that they would have administered histamine (0–10 mM) after treatment with mepyramine or loratadine. This is evidence that students actively reflected upon their practice and considered the importance of experimental design to generate meaningful data. These are essential skills as identified in recommended core curricula.^3^

The student feedback presented herein does have some limitations. Online student evaluation of teaching is known to have a low response,^33^, ^34^, ^35^ which could in future be improved through the use of paper‐based evaluation responses, which have been shown to have higher rates of response.^36^, ^37^ Final‐year undergraduate project students and masters‐level students involved in delivering this class reported that the experience validated their knowledge and gave them clear insights into experimental preparation and design. Although not conducted in this session, it would be informative to sample students' perceptions of using animals in research before and after the session given that 25% of respondents (*n* = 2) reported concerns with “*disturbing*” the animal or concerns with animals in research more broadly. From the educator's perspective, these concerns enable teaching staff to discuss the value and importance of animals in research, as well as the breadth of species used, while signposting to current research and the principles of the 3Rs. In the study presented here, student feedback was qualitative only. However, the implementation of a qualitative and quantitative questionnaire, making use of a Likert scale with specific questions, would generate more comprehensive and informative feedback on this practical class.

These co‐created student‐led drug trials are already being piloted as multi‐institution collaborations with other higher education institutes utilizing *L. variegatus* under blinded conditions and data being collated across these different institutions. This enables students to engage with the wider academic community and highlights the role of collaboration within biomedical research.

### 4.2 *Lumbriculus variegatus* response to drug compounds

Studies have characterized *L. variegatus* as an indicator organism for toxic compounds in aquatic systems^19^, ^20^, ^38^, ^39^, ^40^, ^41^ but very little is known about how *L. variegatus* responds to drug compounds.^18^, ^42^, ^43^

As such, histaminergic signaling in invertebrates appears to be exclusively through ionotropic histamine receptors.^27^ Herein, we demonstrated that 1 mM histamine was capable of inhibiting movement with effects being reversible 24 h after exposure (Figure 2A,B) while exposure to 5 and 10 mM histamine was lethal to *L. variegatus* (Figure 2C,D). Previously, it has been demonstrated that genomic comparison of metabotropic bioamine receptors between vertebrates and invertebrates show no direct homologs within invertebrates to their vertebrate metabotropic histamine receptor counterparts.^27^ It has been reported that in invertebrates histamine activates histamine‐gated chloride channels^27^ and so the administration of histamine may hyperpolarise cells preventing signaling and inhibiting the ability to perform stereotypical movements. Other members of Oligochaeta, such as *Lumbricus terretris*, have been shown to express histamine receptors with a proposed role in innervating the musculature of the body wall.^44^ Moreover, the antihistamine drugs cimetidine and fexofenadine have been shown to inhibit invertebrate growth.^45^, ^46^ Based on the effects of histamine observed in *L. variegatus*, it is likely this organism expresses a histamine receptor homolog and may offer some advantages to other invertebrate species used in biomedical research, namely *Caenorhabditis elegans*, which lacks histamine as an endogenous neurotransmitter.^47^

Mepyramine and loratadine are inverse agonists that competitively antagonize histamine binding and inhibit ligand‐independent signaling from the histamine H_1_ receptor in vetebrates.^48^ Here, mepyramine was shown to inhibit the movement of *L. variegatus* at 1–10 mM without any observable toxicity after exposure (Figure 3). Loratadine has been demonstrated to be toxic to aquatic species within the micromolar range when released via excreta into wastewater.^49^ As such, higher concentrations of loratadine would likely result in significant *L. variegatus* lethality. As with mepyramine, loratadine resulted in the inhibition of stereotypical movements with effects being reversible and returning to a level indistinguishable from pre‐exposure conditions 24 h after exposure (Figure 4).

Administration of histamine and the inverse agonist mepyramine (Figure 5) produced results, which very closely resembled mepyramine treatment alone (Figure 3). However, we did observe a decreased ability to perform stereotypical movements after 1 mM histamine followed by 0.1 mM mepyramine treatment (Figure 5C,D), which we did not observe with mepyramine alone (Figure 3C,D). Loratadine effects were also exacerbated when given 1 mM histamine (Figure 6), which is likely due to the inhibitory effects of 1 mM histamine in isolation as shown in Figure 2.

Interestingly, a delayed effect on helical swimming 10 min after exposure was observed for 1 mM mepyramine (Figure 3D) and 6 μM loratadine (Figure 4D) when given in isolation, but this effect was not seen with body reversal. Previously, copper has been shown to differentially affect the ability of *L. variegatus* to perform stereotypical movements, with helical swimming being more profoundly inhibited.^19^ This may be due to the different nerve fibers responsible for sensing within these two regions; the medial giant fiber is activated following stimulation of the anterior of *L. variegatus* while stimulation of the posterior will activate the paired lateral giant fibers.^19^ When activated, the giant fibers subsequently activate motor neurons and, while the primary neurotransmitter for the medial giant fiber has been proposed to be glutamate,^50^ those regulating the lateral giant fibers have not yet been elucidated. The delayed differential effects observed on helical swimming after treatment with mepyramine (Figure 3D) and loratadine (Figure 4D) may be due to the role of histamine signaling within the lateral giant fibers.

Herein, our findings did not demonstrate the antagonistic effects expected for mepyramine nor loratadine when given in combination with histamine (Figures 5 and6). The effects observed may be due to mepyramine and loratadine acting through off‐target effects resulting in the stereotypical movement inhibition observed (Figures 5 and 6). However, the effects did enable students to reflect on appropriate experiment design. Therefore, further study by genomic analysis and/or immunohistochemistry will be required to further elucidate the presence of histamine receptor homologs within *L. variegatus* and to begin to determine their function in this organism. Currently, it is unknown whether any of the test compounds have a target site within the organism or if the observations seen herein are simply off‐target toxicity affecting *L. variegatus* movement.

Our study presented here provides a novel approach to addressing the in vivo skills gap^8^, ^9^ through co‐created student‐led drug trials using the novel *L. variegatus*. Students gain basic training in in vivo research at a time when animal models for pharmacology education continue to decline^5^ while directly contributing to *L. variegatus* pharmacological research. This method of co‐creation teaching is positively reflected in the student feedback received and provides a more ethical approach to in vivo practical classes compared to conventional in vivo practical classes using mammalian tissues.

## AUTHOR CONTRIBUTIONS

*Participated in research design*: Nia A. Davies, Margaret R. Cunningham, Melisa J. Wallace and Aidan Seeley. *Conducted experiments*: Julanta J. Carriere and Aidan Seeley. *Performed data analysis*: Aidan Seeley. *Wrote or contributed to the writing of the manuscript*: Julanta J. Carriere, Nia A. Davies, Margaret R. Cunningham, Melisa J. Wallace and Aidan Seeley.

## FUNDING INFORMATION

This work was supported by an Education Grant from the British Pharmacological Society.

## CONFLICT OF INTEREST STATEMENT

The authors declare no conflict of interest with the contents of this article.

## ETHICS STATEMENT

*Lumbriculus variegatus* exempt from the Animal (Scientific Procedures) Act 1986 and, therefore, ethical approval was not required for the work presented herein.

## Data Availability

The data that support the findings of this study are available from the corresponding author upon reasonable request.
